# Exploring hematic crasis variations in cancer patients following SARS-CoV-2 vaccination: a real-practice study

**DOI:** 10.1186/s13027-023-00532-9

**Published:** 2023-10-17

**Authors:** Liliana Montella, Carmela Dell’Aversana, Daniela Pacella, Simona Troise, Paola Russo, Valentina Cacciapuoti, Alessandro Ottaiano, Luigi Di Marino, Paola Coppola, Carmela Liguori, Massimiliano Berretta, Salvatore Maddaluno, Lucia Altucci, Gaetano Facchini

**Affiliations:** 1 Oncology Operative Unit, “Santa Maria delle Grazie” Hospital, Pozzuoli, Napoli, ASL NA2 NORD 80078 Italy; 2 Institute Experimental Endocrinology and Oncology “Gaetano Salvatore” (IEOS)- CNR IT, Naples, Italy; 3 Department of Precision Medicine, Università degli Studi della Campania “Luigi Vanvitelli”, Naples, 80131 Italy; 4 Department of Public Health, University of Naples Federico II, Napoli, 80131 Italy; 5 Food and Nutrition Hygiene Service (Servizio Igiene degli Alimenti e della Nutrizione, SIAN), Monteruscello, Pozzuoli, Napoli, 80078 Italy; 6 UOSD cure palliative PO San Gennaro, ASL NA1 Centro, Napoli, 80136 Italy; 7 Department of Laboratory Medicine, Unit of Laboratory of Clinical Pathology, “S.Maria delle Grazie” Hospital, ASL Napoli 2 Nord, Pozzuoli, Napoli, 80078 Italy; 8 Istituto Nazionale Tumori di Napoli, IRCCS “G. Pascale”, Via M. Semmola, Napoli, 80131 Italy; 9 Pineta Grande Hospital, Via Domiziana, km 30/00, Castel Volturno Caserta, 81030 Italy; 10 Department of Clinical and Experimental Medicine, University of Messina, Messina, 98122 Italy; 11 BIOGEM, 83031 Ariano Irpino, Avellino, Italy

**Keywords:** SARS-CoV-2, Vaccine, Neutropenia, Treatment, cancer

## Abstract

SARS-CoV-2 vaccination is strongly recommended, particularly for fragile patients such as those undergoing active oncological treatments. It is crucial to conduct post-marketing surveillance in this patient population. In our study, we conducted a retrospective analysis of real-world data, including 136 patients who received SARS-CoV-2 vaccines and were undergoing anticancer treatments between March 1st and June 30th, 2021. All patients received mRNA vaccines, namely Pfizer-BioNTech’s COMIRNATY (BNT162b2 mRNA) and Moderna’s mRNA-1273 COVID-19 vaccines. We collected blood samples from the patients one week to 10 days before and after vaccine administration to assess full blood count with white cell differentials. Additionally, we monitored serology titers to detect any previous SARS-CoV-2 infection before hospital admission and tracked changes over time. Our findings revealed a significant occurrence of leukopenia following both the first and second vaccine doses among patients receiving chemotherapy and chemo-immunotherapy. Importantly, this effect was independent of demographic factors such as sex, age, and Body Mass Index. In the chemo-immunotherapy treated group, we observed that concomitant immune-mediated diseases were significantly associated with leukopenia following the second vaccine dose. Notably, in healthy subjects, transient neutropenia was recognized as an adverse event following vaccination. The observed lymphocytopenia during SARS-CoV-2 infection, combined with the impact on leukocyte counts observed in our study, underscores the need for larger post-marketing surveillance studies. Despite a treatment delay occurring in 6.6% of patients, the administration of mRNA vaccines did not have a significant impact on the treatment schedule in our series. These findings from a real-world setting provide valuable insights and suggest avenues for further prospective studies to explore potential complex interactions specific to this patient population.

## Introduction

The emergence and spread of the SARS-CoV-2 infection in early 2020 had a profound impact on both the healthcare and economic systems. In Italy, the high number of reported cases and deaths (20,177,910 cases with around 170,000 deaths in July 2022) highlighted the significant public health challenge posed by this pandemic [1]. Consequently, there has been a strong focus on finding effective treatments and promoting widespread SARS-CoV-2 vaccination. The spread of the virus has particularly affected cancer treatment, leading to reduced access to cancer screening, surgical and medical therapies, and increased involvement of healthcare personnel in vaccination efforts. This situation has necessitated symptom management in triage and has even impacted the functioning of oncology departments [2–4].

To address these challenges, on January 2, 2021, the Italian Ministry implemented measures to prevent SARS-CoV-2, including a strategic national vaccine program [5]. In this program, cancer patients were classified as a high-risk group due to their increased susceptibility to severe SARS-CoV-2 infection resulting from their underlying cancer, anticancer treatments, and associated immunodeficiency. This classification was based on recommendations from major Italian health institutions and international cancer management organizations (Agenzia Italiana del Farmaco, National Institute of Health, World Health Organization). In March 2021, four vaccines received approval from the European Medicines Agency (EMA): Pfizer-BioNTech’s COMIRNATY, Moderna’s Spykevax, AstraZeneca’s Vaxzevria, and Johnson & Johnson’s Ad26.COV2.S. These vaccines were recommended for fragile patients at an increased risk of severe SARS-CoV-2 infection, including cancer patients [6–9].

The optimal timing of vaccination in relation to cytotoxic chemotherapy or immunotherapy has not been definitively established. However, it is generally recommended to administer the vaccine toward the end of the therapy cycle to minimize the risk of overlapping with periods of low blood cell count. Vaccine administration should not cause delays in chemotherapy and should avoid coinciding with the nadir period induced by chemotherapy. However, limited information is available regarding the recommended intervals between vaccination and treatment, as well as the impact on hematological parameters following vaccination [10–12].

Our study aims to gather data and information from real-world clinical practice to evaluate the use of SARS-CoV-2 vaccines in cancer patients. Real-world data has proven valuable in uncovering factors that may have been underestimated in clinical studies, despite its limitations. Given the unique nature of mRNA-based SARS-CoV-2 vaccines, which were rapidly developed in response to the pandemic, post-marketing surveillance is highly recommended and expected.

To this end, we conducted a retrospective analysis of hematological parameters following vaccine administration and their potential impact on the scheduling of anticancer intravenous infusions. Additionally, unlike many trials focusing on seroconversion based on anti-Spike (S) antibody titers, we collected data on anti-nucleocapsid (N) COVID-19 antibodies, which are specifically associated with SARS-CoV-2 infection. In summary, our investigation aims to evaluate clinical and hematological parameters in a real-world dataset of cancer patients receiving SARS-CoV-2 vaccines during oncological treatments.

## Patients and methods

### Clinical management and vaccine administration

From January 2021 onwards, all patients with cancer undergoing treatment in our institution were offered two doses of the BNT162b2 mRNA vaccine, administered at a 21-day interval as recommended. Patients with a previous SARS-CoV-2 infection within the past 6 months were excluded from the vaccination program. The timing of vaccine administration was carefully coordinated with the patients’ oncological treatments, taking into consideration optimal intervals to avoid the nadir period induced by chemotherapy. All chemo-, immune- and chemo-immunotherapy protocols were used according to oncological guidelines. Patients were required to have a prednisone dosage lower than 10 mg at least seven days before and after the scheduled vaccine administration.

A retrospective review of data was conducted on patients who received the vaccine between March and June 2021 in the Medical Oncology Unit of S.Maria delle Grazie Hospital. Prior informed consent was obtained from all patients, and their personal data were anonymized in the database. The study protocol was reviewed and approved by the Campania Centro Ethical Committee (Prot. CE n.111 15/04/2022, N.Reg. 20/2022 oss). Out of the 220 screened patients, 136 were included in the analysis after excluding those with missing data, completed oncological treatment before vaccine administration, vaccine administration outside the anticancer treatment schedule, or patient refusal.

Table 1 provides an overview of the general characteristics of the patient population, including information on concomitant diseases, nutritional status (evaluated by Body Mass Index), primary tumor site, cancer stage, type of oncological treatments, and the use of white blood cell growth factors. Routine blood exams were performed before and after both vaccine doses, and white blood cell (WBC) counts were recorded in the database. The BNT162b2 mRNA COVID-19 vaccine was administered, and any adverse events were documented. Leukopenia and neutropenia were defined as an absolute leukocyte and neutrophil count of less than 4000 and 1900 cells/µL, respectively. Normal ranges for lymphocyte count were 0.90–5.20 × 10^3/µL, platelet count 150–400 × 10^3/µL, and hemoglobin level 12.0–18.0 g/dL. Adverse events were graded using the National Cancer Institute’s Common Terminology Criteria for Adverse Events version 5.0.

**Table 1:** Clinical and demographic information of the general cohort

Variable	No. (%)
Gender	
Female	87 (64%)
Male	49 (36%)
Age	64 (11)
Weight (kg)	69 (12)
Height (m)	11 (39)
BMI	25.9 (4.1)
Nutritional status	
normal weight	55 (40%)
obese	21 (15%)
overweight	58 (43%)
underweight	2 (1.5%)
Oncological treatment	
CT	64 (47%)
CT + IO	37 (27%)
IO	35 (26%)
Concomitant disease	97 (71%)
Cardiovascular disease	57 (42%)
Metabolic disease	31 (23%)
Hematologic disease	3 (2.2%)
Immune-mediated	5 (3.7%)
Lung disease	7 (5.1%)
Liver disease	5 (3.7%)
Psychiatric disorders	2 (1.5%)
Allergy to drugs	20 (15%)
Endocrine disease	11 (8.1%)
Early disease	19 (14%)
Advanced disease	117 (86%)
Antibody before vaccine	
Negative	94 (85%)
Positive	17 (15%)
Antibody Titer one-month	
Negative	81 (81%)
Positive	19 (19%)
Antibody Titer at 3-months	
Negative	70 (84%)
Positive	13 (16%)
Antibody Titer at 6-months	
Negative	45 (82%)
Positive	10 (18%)
Growth factors use	
No	114 (84%)
Yes	22 (16%)

### SARS-CoV-2 detection

SARS-CoV-2 detection was performed using the Elecsys® Anti-SARS-CoV-2 assay, which measures total antibodies against the nucleocapsid protein of SARS-CoV-2. IgG and IgM antibodies against SARS-CoV-2 were measured in human serum and plasma. The assay allowed for differentiation between asymptomatic or oligosymptomatic patients and aided in directing them toward confirmatory testing. Data on seroconversion, neutralizing antibodies, and previous values were collected at four-time points: before vaccination, one, three, and six months after vaccination.

### Statistical analyses

Statistical analyses were conducted using descriptive statistics for quantitative and categorical variables. Differences between pre-vaccine and post-vaccine measures were assessed using Student’s t-test for paired samples. Linear regression models were used to investigate predictive factors for significant differences in the WBC count. A significance level of alpha = 0.05 was applied for all analyses, which were performed using R statistical software version 4.0.3.

## Results

Our cohort consisted of 136 enrolled cases, with the majority being breast cancer patients, followed by gastrointestinal, lung, and genitourinary malignancies. Metastatic disease was present in 86% of the patients. Females accounted for 64% of the total patient population. The median age was 66 years (IQR 31–86). Most patients (over 80%) had a normal or overweight classification based on BMI. Comorbidities were present in 65% of cases, with cardiovascular diseases being the most commonly reported. Approximately 50% of patients received chemotherapy, while the remaining 25% received immune therapy or chemo-immunotherapy.

White cell growth factors were administered to 22 out of 136 patients (16%). Regular growth factors were used in only one case, while pegylated growth factors were preferred in the others. In three cases, growth factor support was only provided with one vaccine dose.

Vaccine administration did not result in major side effects in our patient series. The most frequently reported effects (approximately 60% of cases) were local, including a sore arm and local skin reactions. Systemic effects such as fatigue and muscle pain were reported in less than 20% of cases. No grade 3–4 toxicity was recorded.

**Fig. 1:**
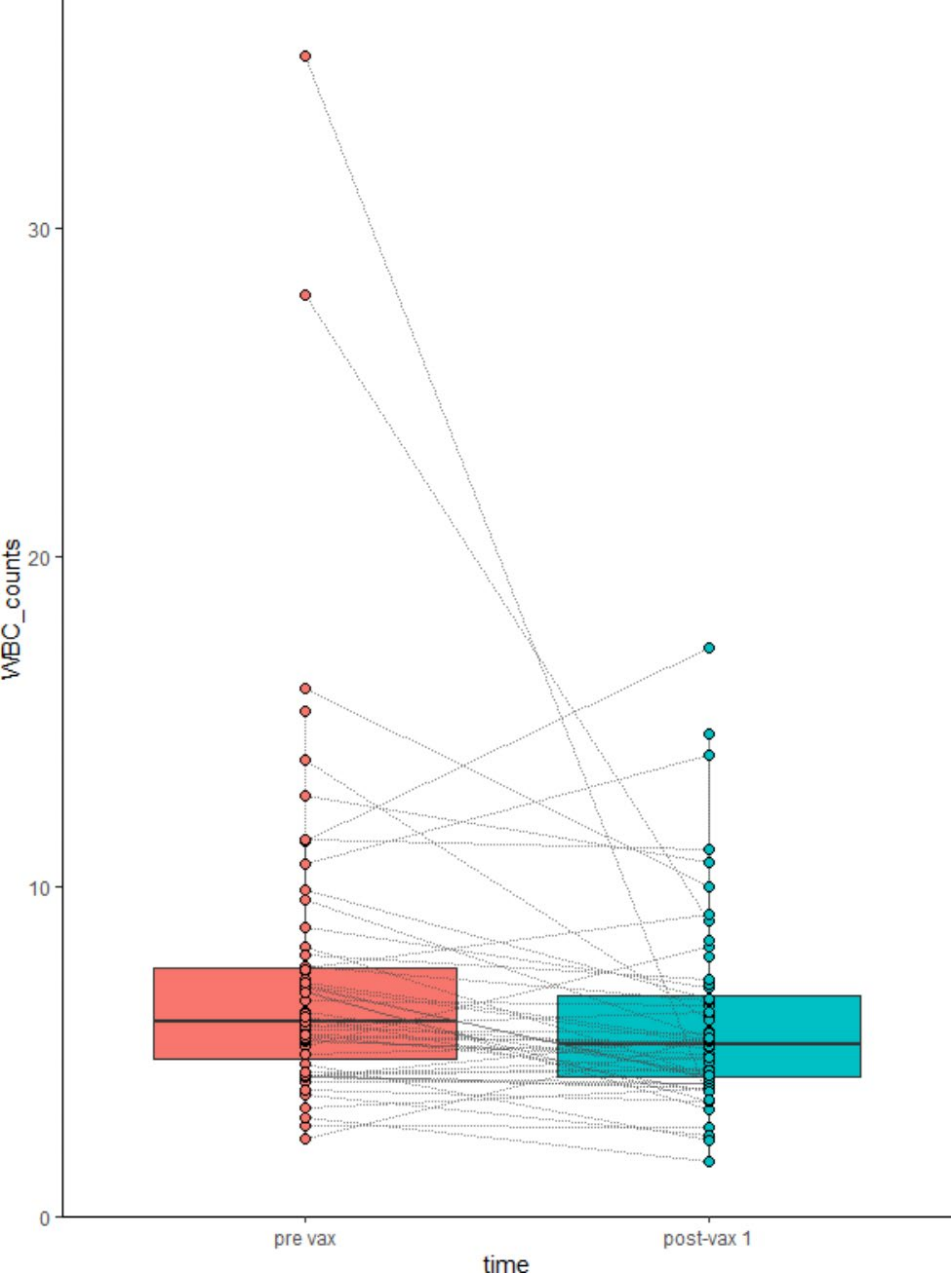
White blood cells counts before and after vaccine administration are represented with two marked outliers

There were no significant differences in hematologic parameters between baseline, post-vax 1 (after the first vaccine dose), and post-vax 2 (after the second vaccine dose) in all patients (Table 2). When analyzing subgroups based on the treatment administered (immunotherapy, referred to as “IO”; chemotherapy, referred to as “CT”; and chemo-immunotherapy, referred to as “IO + CT”), a significantly lower WBC value (P = 0.02841*) was found between baseline and the first vaccine dose in the chemotherapy-treated group (Table 3), but not in the immunotherapy cohort (Table 4). Figure 1 depicts the difference in WBC before and after the first vaccine dose in the chemotherapy group, with two outliers. The linear regression model indicates that this significant difference is not explained by any demographic predictive factors, although having a concomitant immune-mediated disease is a significant risk factor (p < 0.001, estimated WBC count difference coefficient = 15) (Table 5).

**Table 2:** Difference between hematic crasis at baseline, post-vax 1, and post-vax 2 in the general cohort

Variable N = 136	Pre-vax	Post-vax 1	Post-vax 2	Pre-vax vs. Post-vax 1 p-value	Pre-vax vs. Post-vax 2 p-value	Post-vax 1 vs. Post-vax 2 p-value
WBC counts	6.59 (4.18)	6.20 (2.81)	6.40 (3.38)	0.2606	0.5436	0.3893
Neutrophils	4.38 (3.77)	4.02 (2.49)	4.10 (2.97)	0.3515	0.4968	0.8062
Lymphocytes	1.53 (0.77)	1.48 (0.69)	1.55 (0.77)	0.5818	0.8166	0.4277
Platelets	240 (96)	242 (106)	231 (87)	0.8741	0.4226	0.3554
Hemoglobin	12.43 (1.65)	12.40 (1.73)	12.32 (1.68)	0.8814	0.5887	0.7035

**Table 3:** Difference between hematic crasis at baseline, post-vax 1, and post-vax 2 in the cohort under CT treatment

Variable N = 64	Pre-vax	Post-vax 1	Post-vax 2	Pre-vax vs. Post-vax 1 p-value	Pre-vax vs. Post-vax 2 p-value	Post-vax 1 vs. Post-vax 2 p-value
WBC counts	7.4 (5.3)	5.99 (2.94)	6.48 (3.98)	0.02841*	0.1692	0.1604
Neutrophils	5.24 (4.85)	4.01 (2.62)	4.31 (3.57)	0.07737	0.2162	0.5961
Lymphocytes	1.45 (0.60)	1.32 (0.48)	1.43 (0.63)	0.1917	0.8856	0.2677
Platelets	245 (112)	238 (105)	238 (104)	0.7165	0.684	0.9649
Hemoglobin	12.17 (1.62)	12.09 (1.69)	12.09 (1.68)	0.8038	0.7891	0.9862

**Table 4:** Difference between hematic crasis at baseline, post vax 1 and post vax 2 in the cohort under IO treatment

Variable N = 35	Pre-vax	Post-vax 1	Post-vax 2	Pre-vax vs. Post-vax 1 p-value	Pre-vax vs. Post-vax 2 p-value	Post-vax 1 vs. Post-vax 2 p-value
WBC counts	6.15 (2.39)	6.38 (2.37)	6.21 (2.02)	0.4804	0.8602	0.4156
Neutrophils	3.71 (2.11)	3.80 (2.02)	3.55 (1.46)	0.8526	0.7188	0.5564
Lymphocytes	1.75 (0.88)	1.75 (0.86)	1.85 (0.96)	0.9792	0.6535	0.6691
Platelets	235 (68)	250 (127)	222 (52)	0.5414	0.3934	0.2433
Hemoglobin	13.22 (1.29)	12.97 (1.41)	13.02 (1.49)	0.445	0.556	0.8825

**Table 5:** Simple linear regression model of factors predicting difference in WBC count pre-vax vs. post-vax 1 in CT-treated group

Characteristic	Difference between WBC count pre- vs. post-vax 1 in the CT group			
	N	Beta	95% CI	p-value
Gender	63			0.461
Female		—	—	
Male		-1.0	-3.6, 1.7	
Age	63	-0.03	-0.14, 0.08	0.606
BMI	63	-0.14	-0.46, 0.17	0.372
Concomitant disease	63			0.637
No		—	—	
Yes		0.66	-2.1, 3.5	
Cardiovascular disease	63			0.549
No		—	—	
Yes		-0.78	-3.4, 1.8	
Metabolic disease	63			0.794
No		—	—	
Yes		-0.43	-3.7, 2.8	
Hematologic disease	63			0.627
No		—	—	
Yes		2.5	-7.8, 13	
Immune-mediated	63			< 0.001
No		—	—	
Yes		15	9.1, 21	
Lung disease	63			0.817
No		—	—	
Yes		0.55	-4.2, 5.3	
Liver disease	63			0.938
No		—	—	
Yes		-0.24	-6.3, 5.8	
Allergy to drugs	63			0.904
No		—	—	
Yes		-0.27	-4.6, 4.1	
Endocrine disease	63			0.950
No		—	—	
Yes		-0.23	-7.6, 7.1	
Advanced disease	63			0.225
No		—	—	
Yes		-2.3	-6.2, 1.5	
Antibody before vaccine	56			0.412
Negative		—	—	
Positive		-1.6	-5.5, 2.3	
Antibody Titer one-month	52			0.880
Negative		—	—	
Positive		-0.18	-2.5, 2.2	
Antibody Titer at 3-months	35			0.945
Negative		—	—	
Positive		-0.07	-2.0, 1.9	
Antibody Titer at 6-months	27			0.485
Negative		—	—	
Positive		-1.4	-5.4, 2.6	
Growth factors use	63			0.064
No		—	—	
Yes		2.5	-0.15, 5.2	

The immune-mediated diseases associated with cancer in our patient series included neurologic syndromes such as Lambert-Eaton and multiple sclerosis, inflammatory bowel diseases like Crohn’s disease, rheumatic polymyalgia, and systemic lupus erythematosus.

Only the subgroup of patients treated with chemo-immunotherapy showed a significantly lower WBC value (P = 0.02427*) between the baseline and the second vaccine dose (Table 6). Once again, the linear regression model indicates that the lower WBC value in patients treated with CT-IO regimens is not related to any of the considered predictive factors (Table 7). No significant changes were found in lymphocyte subpopulations, hemoglobin, or platelet counts.

**Table 6:** Difference between baseline, post-vax 1, and post-vax 2 in the cohort under IO + CT treatment

Variable N = 37	Pre-vax	Post-vax 1	Post-vax 2	Pre-vax vs. Post vax1 p-value	Pre-vax vs. Post-vax 2 p-value	Post-vax 1 vs. Post-vax 2 p-value
WBC counts	5.66 (3.00)	6.4 (3.0)	6.45 (3.33)	0.2219	0.02427*	0.9323
Neutrophils	3.52 (2.31)	4.24 (2.70)	4.26 (2.89)	0.228	0.2295	0.9673
Lymphocytes	1.46 (0.90)	1.49 (0.76)	1.47 (0.74)	0.8648	0.9462	0.9089
Platelets	235 (89)	240 (85)	227 (84)	0.8058	0.7094	0.5255
Hemoglobin	12.12 (1.80)	12.37 (1.96)	12.04 (1.72)	0.5722	0.8438	0.4449

**Table 7:** Simple linear regression model of factors predicting difference in WBC count pre-vax vs. post-vax 2 in IO + CT-treated group

Characteristic	Difference between WBC count pre-vax vs. post-vax 2 in the IO + CT group			
	N	Beta	95% CI	p-value
Gender	37			0.299
Female		—	—	
Male		0.80	-0.74, 2.3	
Age	37	0.03	-0.04, 0.11	0.381
BMI	37	-0.05	-0.21, 0.12	0.552
Concomitant disease	37			0.867
No		—	—	
Yes		0.15	-1.6, 1.9	
Cardiovascular disease	37			0.385
No		—	—	
Yes		0.60	-0.79, 2.0	
Metabolic disease	37			0.937
No		—	—	
Yes		0.07	-1.6, 1.8	
Hematologic disease	37			0.573
No		—	—	
Yes		1.2	-3.1, 5.5	
Immune-mediated	37			0.839
No		—	—	
Yes		0.31	-2.8, 3.4	
Lung disease	37			0.130
No		—	—	
Yes		-3.2	-7.3, 1.0	
Liver disease	37			0.553
No		—	—	
Yes		0.90	-2.2, 4.0	
Allergy to drugs	37			0.835
No		—	—	
Yes		-0.17	-1.8, 1.4	
Endocrine disease	37			0.258
No		—	—	
Yes		1.1	-0.86, 3.1	
Advanced disease	37			0.737
No		—	—	
Yes		-0.37	-2.6, 1.9	
Antibody before vaccine	31			0.706
Negative		—	—	
Positive		0.51	-2.2, 3.3	
Antibody Titer one-month	25			0.553
Negative		—	—	
Positive		0.73	-1.8, 3.3	
Antibody Titer at 3-months	22			0.988
Negative		—	—	
Positive		-0.02	-2.8, 2.7	
Antibody Titer at 6-months	9			0.515
Negative		—	—	
Positive		1.1	-2.7, 5.0	
Growth factor use	37			0.193
No		—	—	
Yes		2.7	-1.4, 6.9	

There was no significant association between the use of growth factors and white blood cell counts before and after vaccine administration (Table 5). Among the 136 patients, 9 (6.6%) experienced a delay in treatment administration, with two of them receiving pegylated growth factors. Three patients were receiving combined chemo-immunotherapy. In only one case, the reported delay affected both vaccine doses.

Baseline anti-N antibody titers were available for 111 out of 136 patients, and at least two measurements were available for 105 patients. In our cohort, 17 patients (15% of the cohort, as shown in Table 1) exhibited positive values (COI > 1). Among the 111 patients, 14 (12%) had a COI higher than 20 before vaccine administration, while three had a COI lower than 20. In all cases except for five without reported infections, there was concordance with a previous SARS-CoV-2 infection before vaccination. However, two of these cases had a COI < 20. Additionally, one patient reported an overt SARS-CoV-2 infection with mild symptoms eight months after receiving the second vaccine dose.

## Discussion

In 2023, although the attention on SARS-CoV-2 has diminished, the state of emergency we experienced and the urgent need to address the increased risk of death in cancer patients through preventive measures and vaccination campaigns have provided valuable learning experiences. Our retrospective study aims to describe the variables of interest in a cohort exclusively composed of patients undergoing anticancer therapy. It would be highly interesting to conduct future comparative analyses between healthy subjects and the oncological population undergoing active treatments to assess the impact of the disease and anticancer regimens compared to a baseline condition.

Cancer patients face a significantly higher risk of severe SARS-CoV-2 infection. Previous reports have indicated a mortality rate ranging from 5 to 30% in these patients [13–15]. Several factors contribute to the increased risk of death from SARS-CoV-2, including cancer- and treatment-related immunosuppression, hematological malignancies, particularly lung cancer, and non-cancer-related factors such as advanced age, metabolic disorders, and cardiovascular diseases. Endocrine-related diseases, which require specialized management during anticancer treatments and SARS-CoV-2 infection, also play a significant role, accounting for approximately 8% of cases in our series. The interaction between SARS-CoV-2 and existing endocrine dysfunctions can worsen the overall prognosis. Therefore, proper preventive measures and close attention are crucial, particularly during oncological (immune) treatments known to impact endocrine function [16].

Considering the high risk faced by cancer patients, the administration of SARS-CoV-2 vaccines is strongly recommended. While pre-marketing clinical trials provide limited data, an increasing amount of real-world evidence supports the safety and effectiveness of these vaccines, including the development of antibody responses.

In our study, we report the effects of SARS-CoV-2 vaccine administration on blood cells and the potential impact on oncological treatment in a well-characterized cohort of cancer patients. Most studies have primarily focused on safety and seroconversion, with few providing incidental reports regarding other outcomes. In our cohort, approximately 6% of patients experienced delays in treatment administration. We found a significant association between the administration of chemotherapy after the first vaccine dose and combined chemo-immunotherapy after the second dose with leukopenia. However, no statistically significant effects were observed in the general cohort of patients or those receiving immunotherapy alone. Furthermore, no significant alterations were noted in lymphocyte subpopulations, hemoglobin levels, or platelet counts.

Differential effects of various oncological treatments on white blood cells may explain these findings. Neutropenia, for instance, is predominantly observed 7–12 days after chemotherapy administration, while combined regimens may have a more delayed effect. An interesting report indicates a potential positive prognostic value of a single episode of neutropenia in lung cancer patients treated with chemo-immunotherapy, suggesting a reduced inhibitory effect on T-cells by suppressor neutrophils [17]. Several recognized risk factors, including age, low body mass index, baseline white blood cell counts, disease stage, and treatment lines, contribute to the development of neutropenia during oncological treatments. Notably, our cohort exhibited significant leukopenia but no significant neutropenia in correlation with chemotherapy and chemo-immunotherapy. Age, sex, and BMI did not show a statistically significant association with leukopenia, while immune-mediated diseases were significant predictors.

In a comparative evaluation conducted in Israel, a group of 232 cancer patients receiving various anticancer treatments and vaccinated with the SARS-Cov-2 BNT162b2 vaccine, along with 261 healthy subjects, reported a leukopenia rate of 39% among seronegative patients, without further details [7]. The prospective multicenter VOICE trial using the mRNA-1273 vaccine on patients with solid tumors undergoing chemotherapy, immunotherapy, or chemoimmunotherapy reported only one case of febrile neutropenia in the chemotherapy cohort, without providing data on the incidence of every grade of neutropenia [18].

Another study assessing the effects of the BNT162b2 vaccine in 154 cancer patients with solid tumors compared to a control group documented a delay in anticancer treatment in nine (6%) patients, primarily due to neutropenia (7 out of 9 patients) [19]. However, only a single episode of treatment delay was reported, and overall administration schedules were largely maintained. The incidence reported in this study aligns well with the delay observed in our cohort.

A recent study specifically focused on hematological abnormalities following the administration of inactivated whole-virion SARS-CoV-2 vaccine (CoronaVac, Sinovac) and the mRNA vaccine BNT162b2 in healthy subjects reported an increased risk of leukopenia shortly after the second dose of BNT162b2 [20]. Similarly to our study, a significantly decreased leukocyte count, rather than neutrophils, was found. The authors hypothesized that leukopenia was due to reduced lymphocyte counts, but they could not support this hypothesis due to the unavailability of lymphocyte and WBC count data. In contrast, our study included these hematological parameters, but the small and heterogeneous patient sample size prevented the identification of statistically significant decreases in lymphocyte or neutrophil counts. Therefore, no conclusive evidence can be drawn regarding the specific white blood cell types involved in lower leukocyte counts. The reported global incidence of hematological abnormalities after SARS-CoV-2 vaccination ranges from 0.2 to 2.5 cases per 10,000 vaccine doses. Particularly, the study by Sing et al. observed an increased risk of leukopenia following the second dose of BNT162b2 [20]. Although subjects with cancer were not included in their study, Sing et al.‘s data support the presumed causal role of the SARS-CoV-2 vaccine in inducing temporary neutropenia. Food and Drug Administration Philippines received reports concerning hematological events [21], thus, raising interest in this matter. A case-controlled series coming from the national Philippines database and including children and adults reported on 268 individuals out of a total of 146,839,247 vaccine doses administered highlights that hematological events can be registered at a low rate without sequels and with confirmed safety [22]. In addition, another informative paper on 187 patients reports that following the second vaccine dose the neutrophil-to-lymphocyte ratio (NLR) was not significantly different in vaccinated patients versus non-vaccinated COVID-19 negative patients [23].

The variations in peripheral blood cell counts can be influenced by various factors, including the concurrent use of medications such as antiretrovirals, corticosteroids, antibiotics, and the presence of concomitant viral infections. In cancer patients undergoing chemotherapy, the use of granulocyte growth factors can also affect these variations. In our study, corticosteroids were gradually reduced before vaccination to potentially enhance seroconversion. White blood cell growth factors were used according to guidelines for high neutropenic regimens. However, the use of growth factors did not prevent the decrease in white blood cell counts, as two out of nine patients with treatment delays received pegylated factors.

Lymphocytopenia, a reduction in lymphocyte counts, is commonly observed during SARS-CoV-2 infection and is considered a poor prognostic factor [24, 25]. The interaction between the SARS-CoV-2 virus and lymphocytes is mediated through the Spike protein. We hypothesize that the vaccine-induced reaction, which characteristically leads to hypermetabolic lymph nodes [26] and potential drainage of lymphocytes, may contribute to the relative reduction of peripheral white blood cells [7]. Redistribution of white blood cells throughout the body has been documented after vaccine administration, ranging from approximately 14% to more than 50% [27], particularly highlighted after the SARS-CoV-2 vaccine. This redistribution has raised challenges in interpreting imaging results. A recent study reported 44% lymphopenia among 260 patients who underwent 18 F-FDG PET/CT scans [26]. The study found an inverse relationship between SARS-CoV-2 vaccine-induced hypermetabolic lymph nodes and lymphopenia, with the hypermetabolic pattern being more frequently associated with the absence of lymphopenia and possibly indicating a stronger immune response to the vaccine. This observation was independent of specific treatments, with 41% of the population being treatment-free and the others receiving various therapies including chemo-, immuno-, and targeted therapy.

Considering the significantly low leukocyte counts observed in patients receiving chemo- and chemo-immunotherapy, it is plausible to hypothesize that the vaccine and administered treatment have at least an additive effect. Previous studies have also highlighted a reciprocal bidirectional effect exerted by vaccination and immunotherapy [27]. The immune system is a complex network involving specialized cell populations and products, and its regulation occurs at epigenetic, genetic, and protein levels. Defects can occur in immune cells or their progenitors, leading to cancer development or an immune evasive phenotype that establishes an immune suppressive microenvironment. Therefore, we speculate on the potential reciprocal benefits of combining SARS-CoV-2 vaccination with immunotherapy in cancer patients. Further evaluation is warranted based on findings from this retrospective study.

Guidelines in oncology have recommended the use of growth factors to reduce the risk of febrile neutropenia when the risk exceeds 20% [28]. While most recommendations support this practice to minimize the risk of infection [29, 30], concerns have been raised regarding increased neutrophil extracellular traps, elevated levels of inflammatory cytokines, and the potential excess risk of thrombosis. In line with these considerations, the administration of prophylactic granulocyte colony-stimulating factor (G-CSF) should consider the increased risk of an inflammatory state and suggests the cautious use of short-acting G-CSFs [31–33]. Additionally, the administration of chemotherapy can influence the seroconversion induced by the vaccine, with poorer seroconversion observed when the interval between chemotherapy and vaccination is less than 15 days [34].

Serological testing provides valuable information about the immune response to SARS-CoV-2 following natural infection and vaccination. Most studies have used antigen S tests for assessing vaccine-induced immune response [7, 8]. On the other hand, tests based on anti-N antigen provide information about natural infection in vaccinated individuals. Detection of anti-N antibodies following vaccination is considered indicative of encountering the virus. A wide spectrum of cut-off index (COI) values has been observed in asymptomatic, mildly symptomatic, and severely symptomatic SARS-CoV-2 infected patients [35].

The S1 viral subunit plays a crucial role in binding to functional ACE2 receptors on susceptible human cells, enabling the virus to enter these cells. Blocking the virus’s entry through anti-spike antibodies significantly contributes to virus neutralization. Traditionally, higher levels of neutralizing antibodies targeting the spike protein of SARS-CoV-2 have been associated with greater vaccine-induced protection. However, with the increasing prevalence of spike protein mutations in variants, the induction of neutralizing antibodies against the N-protein may also be relevant for maintaining protection. The role of anti-N antibodies in conferring long-term immunity in individuals infected with the virus is still unknown [36]. Due to the retrospective nature of our study, we did not evaluate the anti-S response in this patient cohort.

In conclusion, although our study is limited by a relatively small sample size, it provides insights into the hematological changes following mRNA vaccines in patients with solid cancers undergoing active oncological treatments. Importantly, our findings suggest that the administration of mRNA vaccines does not compromise the scheduled delivery of oncological treatments. Despite its limitations, this study contributes to the growing body of evidence supporting the safe and effective use of mRNA vaccines in this specific patient population.

## Data Availability

All data supporting the findings of this study are available within the paper and refer to an anonymized dataset available in our electronic archive and accessible upon request.
